# Positive nutritional selection of adults with healthy lifestyle and high daily fiber consumption for the isolation of beneficial intestinal bacteria: The iTARGET cohort study protocol

**DOI:** 10.1016/j.mex.2025.103268

**Published:** 2025-03-17

**Authors:** Aurélie Caille, Chloé Connan, Noelle Lyon Belgy, Elise Borezée, Claire Cherbuy, Nathalie Meunier, Victoria Meslier

**Affiliations:** a Centre Hospitalier Universitaire de Clermont-Ferrand, CRNH Auvergne, Clermont-Ferrand, France; b Université Paris-Saclay, INRAE, MetaGenoPolis, 78350 Jouy-en-Josas, France; c Université Paris-Saclay, INRAE, Micalis, 78350 Jouy-en-Josas, France

**Keywords:** Observational cohort, Gut microbiota, Nutrition, Shotgun metagenomics, Culturomics, Next-generation probiotics

## Abstract

Recent advances in the study of the gut microbiota has pointed to its under-utilized source of potentially beneficial bacteria, known as next generation probiotics, offering a promising avenue to restore or compensate impaired gut microbiota toward a healthy state. Aside from the difficulties to achieve in-lab adequate culture conditions, the use of beneficial bacterial isolates is also limited by their bioavailability in the donor itself. In the iTARGET study, we positively selected donors based on their diet enriched in fiber, that has been shown to increase the prevalence of bacterial species associated with health.

The iTARGET study is a monocenter, prospective, observational study of adults with healthy lifestyle and high daily fiber consumption. We aim to recruit individuals in two phases, the first one for all individuals that will permit the identification of carriers for bacteria of interest and the second phase for a subset of individuals to allow for culture and isolation of previously identified potentially beneficial bacteria. Our primary outcome is the isolation and culture of at least one potentially beneficial isolate. The secondary outcomes comprised the high throughput metagenomic profiles of the intestinal microbiota and the characterization of the cultured isolates.

The study was approved by the French Research Ethics Committees (Comité de Protection des Personnes Sud-Est I) under the National reference ID 2023-A01677–38. Study findings and results will be published in peer-reviewed Open Access journals. (Trial registration number on ClinicalTrials.gov: NCT06166810).

## Graphical abstract

**Figure:**
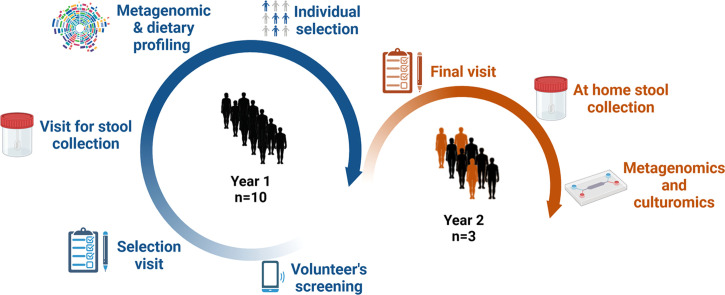

Specifications table

**Table:** 

Subject area:	Food Science
More specific subject area:	Positive selection of healthy donors with high fiber diet consumption
Name of your protocol:	iTARGET study protocol
Reagents/tools:	Not applicable
Experimental design:	The iTARGET cohort is a monocenter, prospective, observational study of adults with healthy lifestyle and high daily fiber consumption.
Trial registration:	NCT06166810
Ethics:	The study was approved by the French Research Ethics Committees (Comité de Protection des Personnes Sud-Est I) under the National reference ID 2023-A01677–38. Written Informed consent will be obtained from each participant before enrollment.
Value of the Protocol:	• This study is based on the positive selection of donors with healthy lifestyles and fiber-rich dietary profiles to provide stools for which we aim to isolate potentially beneficial bacteria. • The collection of stools associated with dietary profiles and omics profiles will provide a unique bioresource for the isolation of potentially beneficial bacteria. • The culture of isolates will permit the definition of optimized conditions to achieve their in vitro cultivation for a wide range of potentially beneficial bacteria and the characterization of the isolates will permit to validate their interest as potentially beneficial bacteria.

## Background

Over the past decades, numerous studies have been performed to gain a better understanding on the role of the intestinal microbiota in human health [1,2]. The gut microbiota, that is the complex and dynamic set of microorganisms colonizing our gastrointestinal system, have been shown to play a crucial role in health and disease, including human digestive, metabolic, immune and neurological functions [3,4]. An imbalance of the gut microbiota, known as dysbiosis, is frequently described in chronic diseases or metabolic dysfunctions in humans [[5], [6], [7]]. In many pathologies, this qualitative or quantitative alteration of the gut bacteria is responsible for its detrimental effect on anti-inflammatory bacterial species, often concomitant to the rises of pro-inflammatory species, ultimately modulating the structure and functionalities of the gut microbial community. Therefore, there is a major interest to directly exploit potentially beneficial bacteria as new-generation probiotics, and/or explore their functionalities, in order to restore or compensate impaired alteration of the gut microbiota toward a healthy state [8,9].

Among the factors influencing this intestinal balance, diet plays a predominant role [[10], [11], [12], [13]]. Mounting evidence from epidemiological studies have highlighted the positive impact of a diet rich in plant sources on human health, including obesity, type 2 diabetes or population at risks of cardiovascular diseases [[14], [15], [16], [17]]. More specifically, the consumption of fruit, vegetables, pulses and whole grain cereals is associated with health and, in particular, with an increase in the relative abundance of potentially beneficial bacterial species [10]. In this context, the modulation of the intestinal microbiota directly via the diet, by providing specific substrates to certain bacterial groups, or directly through the intake of these beneficial bacteria, the so-called next generation probiotics, is therefore a possible promising approach to the prevention and/or treatment of pathologies.

To date, a large number of bacterial species from the intestinal microbiota have yet to be isolated and cultured, limiting the characterization of their potential health benefits. The difficulties in isolating and culturing bacteria from the intestinal microbiota are linked to (i) the optimal culture conditions that are generally unknown and the continuity of the anaerobic conditions, (ii) the methods for isolating bacteria, generally relying on non-targeted approaches and undefined media, and (iii) the strategies for selecting the donors. Bacterial species positively associated with health have been identified using *in silico* metagenomic analysis by comparing healthy individuals and patients confronted to their respective diet [18,19]. In order to isolate and cultivate these beneficial bacteria, individuals with diet enriched in fiber are therefore particularly relevant. In this context, a positive selection of donors based on their dietary profiles is essential to favor the prevalence of these beneficial bacteria. To the best of our knowledge, no study has yet used positive donor selection to isolate new bacterial species with healthy potentials and associated to the consumption of fiber.

### Description of protocol

**Study aims**

**Fig. 1:**
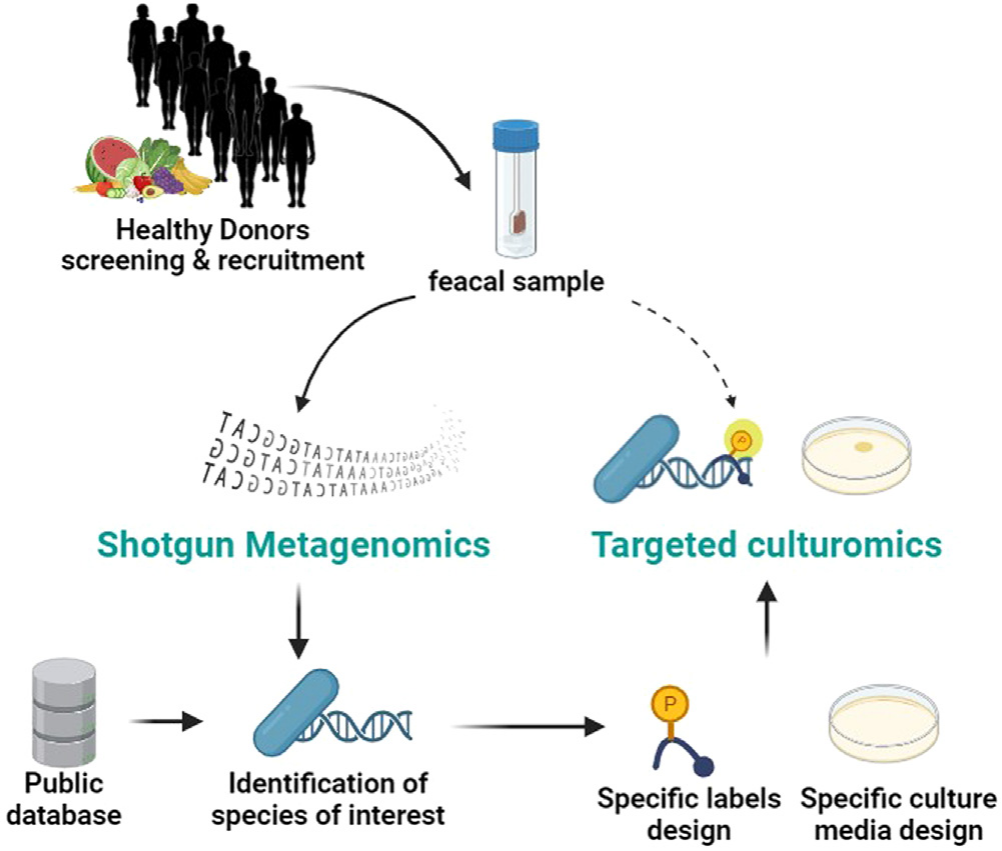
Overall strategy of the iTARGET study.

The primary aim of the study is to isolate bacteria from stools of participants on a high-fiber diet, comprising new isolates of known species (list of selected species in Table 1) or new species for which no isolates have yet been cultured (Fig. 1). The secondary objectives of the study are (i) to characterize the microbiota of the recruited participants in order to identify carriers for bacteria of interest in relation to their diet, and (ii) to characterize isolated bacteria at the phenotypic and genotypic levels.

**Table 1:** Selected bacteria of interest for their isolation and culture in the iTARGET study [20].

Bacterial strain (GTDB taxonomy)	NCBI accession number	Interest	References
*Akkermansia muciniphila* DSM 22,959	GCF_000020225.1	Metabolic syndrome	[21,22]
*Bifidobacterium adolescentis* DSM 20083T	GCF_000010425.1	Intestinal barrier	[23]
*Bifidobacterium bifidum* NCTC 13,001	GCF_900,637,095.1	Colitis	[24]
*Bifidobacterium longum* spp.	GCF_000020425.1 and GCF_900,637,335.1	Gut-brain axis	[25]
*Phocaeicola vulgatus* ATCC 8482	GCF_000012825.1	Diet induced hepatic steatosis	[26]
*Bacteroides intestinalis* DSM 17,393	GCF_000172175.1	Primary polysaccharides degrader	[27]
*Bacteroides fragilis* NCTC 9343 (EN-2)	GCF_000025985.1	Primary polysaccharides degrader	[28]
*Bacteroides thetaiotaomicron* DSM 2079T	GCF_000011065.1	Primary polysaccharides degrader	[28]
*Bacteroides xylanisolvens DSM 18836T*	GCF_000273315.1	Pasteurized milk product fermented with B.xylanisolvens approved as novel food	[29]
*Bacteroides uniformis* ATCC8492	GCA_000154205.1	Post-antibiotic recovery	[30]
*Anaerostipes hadrus* BPB5	GCF_001998765.1	Butyrate producer	[31]
*Anaerostipes caccae* DSM 14,662	GCF_000154305.1	Food allergy	[32]
*Coprococcus eutactus* ATCC 27,759	GCF_000154425.1	Colitis	[33]
*Coprococcus catus* AF45–17	GCF_003433855.1	Short Chain fatty acid producer	[34]
*Coprococcus comes* ATCC 27,758	GCF_000155875.1	Chronic musculoskeletal pain(fibromyalgia)	[35]
*Roseburia intestinalis* DSM 14,610 (L1–82)	GCF_000156535.1	Polysaccharides degraders	[36]
*Roseburia inulinivorans* 2789STDY5608835	GCF_001406675.1	Metabolic syndrome	[36]
*Butyrivibrio crossotus* DSM 2876	GCF_000156015.1	Healthy diet positive association	[37]
*Ruminococcus bromii* L2–36	GCF_002834165.1	Food allergy	[38]

### Study design

The iTARGET cohort is a monocenter, prospective, observational study of adults with healthy lifestyle and high daily fiber consumption. The recruitment period starts September 2023 and ends December 2024.

### Eligibility criteria

Volunteers will be selected from adults individuals, with a healthy lifestyle, i.e. a state considering both diet and physical activity, and consuming (i) a daily fiber consumption of >25*g* per day (a value comprised between the recommendations of the French Agency for Food, Environmental and Occupational Health & Safety (ANSES) of 30*g*/day [39] and the current daily consumption of the French population of 20*g* fiber per day (INCA3 study) [40]) (ii) a daily variety of fiber following the recommendations of the French National Nutrition and Health Program (Programme National Nutrition Santé PNNS), consisting of >5 portions of fruit and vegetables per day [41] and (iii) in compliance with the eligibility criteria defined below.

### Inclusion criteria

• Healthy adult individuals
• Age: ≥ 20 and ≤ 50 years old
• BMI: > 18.5 and < 25 kg/m^2^
• Intake of at least 25*g* of dietary fiber per day
• Consumption of >5 portions of fruit and vegetables a day
• A diversified diet low in ultra-processed foods

### Non-inclusion criteria

• Known pathologies
• Current treatment
• Antibiotics and/or transit modulators taken 3 months prior to sampling
• History of digestive surgery having an impact on the microbiota incompatible with the study (bariatric surgery)
• Consumption of food supplements based on protein preparations, probiotics or prebiotics
• Smoking
• Alcohol consumption: > 2 glasses (i.e. 20 g) per day
• Sedentary lifestyle (< 600 Mets/min/week) or intense sport activity (≥ 3000 Mets/min/week)
• Pregnant or breast-feeding women
• Persons under legal protection (curatorship, guardianship, etc.)
• Volunteers taking part to interventional study
• Person refusing to be registered on the National Register of Healthy Volunteers of the Ministry of Health (VRB)
• Subject not affiliated to the French social security system
• Subject in a period of exclusion from a previous study or having received a total amount of 6000 euros over the 12 consecutive months preceding the start of the trial

### Participants’ recruitment

**Fig. 2:**
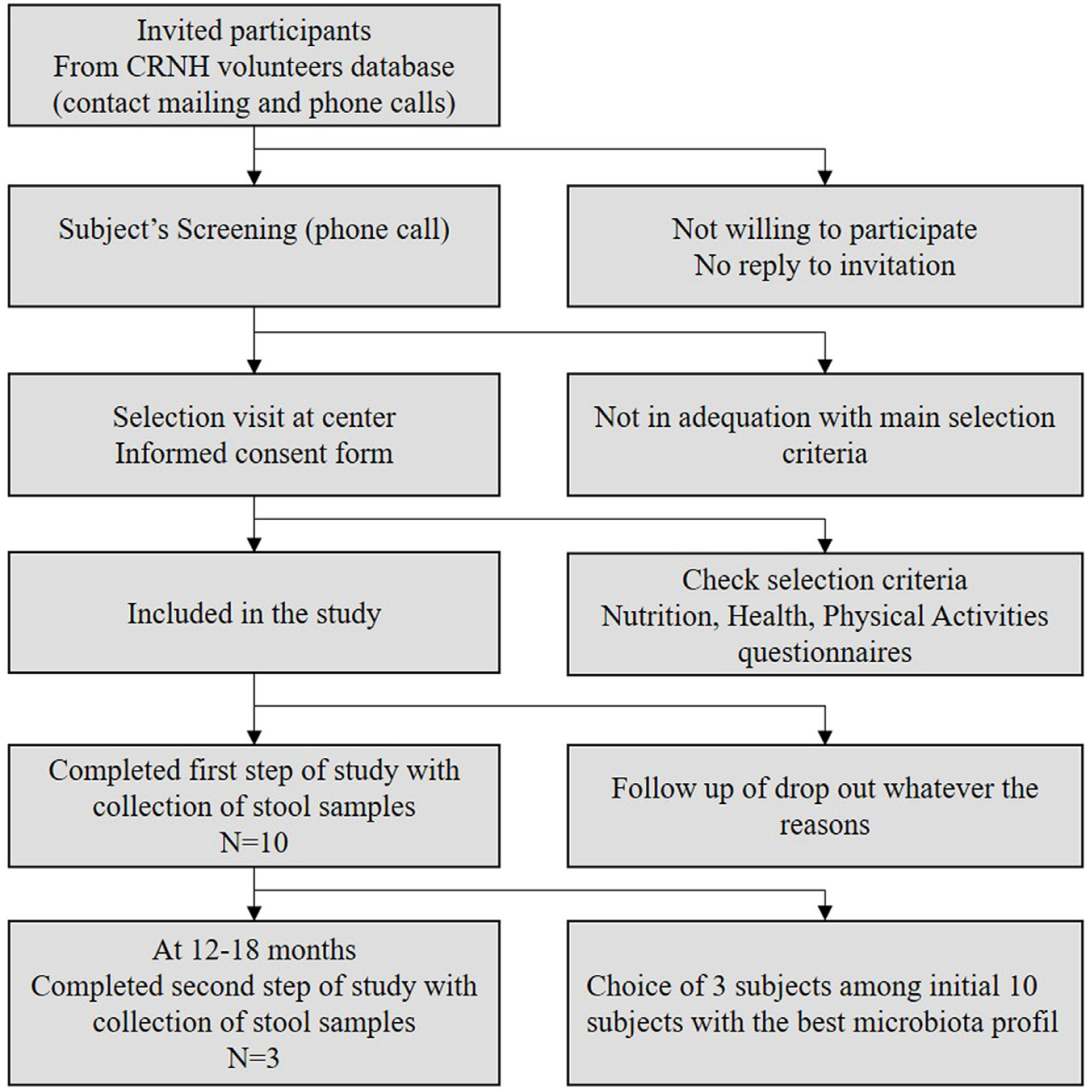
Flow Chart of the participant selection process in the iTARGET study.

At the time of enrollment, after informed consent, subjects will visit the Unité d'Exploration en Nutrition (UEN) center twice or three times, first for a screening visit, a second visit to collect stool samples and complete questionnaires, and a third visit, only for 3 subjects, 12–18 months after the first stool collection as described below (Fig. 2).

To take part of the study, volunteers must complete the participant journey and collect a compliant stool sample. The study will be carried out in two phases. In the first phase (T0), stools from healthy participants will be collected, with a diversified diet rich in fiber, validated by a questionnaire carried out during the selection visit. Each participant will make one stool donation. This collection will permit the identification of carriers for bacteria of interest in relation to their diet and the development of targeted culturomics tools. In a second phase (T0+1 year), individuals selected according to their microbial profiles (*n* = 3) obtained from shotgun metagenomics at T0 in relation to their diet and whose stools presented the bacteria of interest (Table 1) will be recalled for a new stool donation in order to carry out culturomics using tools designed during the first phase (Fig. 1).

### Screening of participants

Subjects will be recruited from the UEN - CRNH Auvergne volunteer's database (declared under the number N°1,080,650 to the "Comité National de l'Informatique et des Libertés", CNIL), by advertisement in the local media and on the CRNH website and the INRAE local media. Subjects will be screened by telephone calls, during which an initial explanation of the study and the protocol will be given. For interested individuals, the information note and consent form will be sent or given to them before the selection visit.

### Selection visit

During the selection visit, the protocol will be explained in detail to the subjects based on the information form by one of the investigators of the UEN center, who will obtain their written agreement by signing the consent form. The subjects will then have their anthropometric parameters measured, and will complete a health and physical activity questionnaires [42], and a Bristol scale form. An interview with a dietician will follow to check the dietary selection criteria, using a dietary questionnaire. If the volunteer is included, he or she will leave with stool collection kits for home sampling. They will also receive a 3-day dietary record and a Bristol scale form to be filled at the time of their stool donation.

### Stool collection visit

During this visit, the subjects will come back at the UEN center, they will provide stool samples they have collected at home and the completed questionnaires. The dietician will check the dietary record with the volunteer and collect the completed Bristol scale. The volunteer will be reminded that he or she may be contacted again for a second stool collection, approximately one year after the first collection, to allow time for the analyses to be carried out on the first stool samples.

### Final visit at 12–18 months for three selected subjects

Based on metagenomic analyses and dietary profiles of the samples from the first phase (T0), three subjects will be recalled for a new stool collection. The subjects will be selected based on their most distinct metagenomic profiles and the detection of the selected species of interest (Table 1). The selection criteria will be checked again for each selected volunteer. The participants will again complete the questionnaires used for recruitment. If the subjects still meet the selection criteria, new stool collection kits will be distributed to them.

Samples that do not pass the initial quality control (e.g. presence of identification data on tube, missing sample, broken tube, etc.) are flagged up and a new sampling is given. QC-validated samples are stored at −80 °C at the MetaGenoPolis-SAMBO BRC (Biological Resources Centre), which is ISO9001 certified and labelled GIS-IBiSA (CRB N°242), until further processing.

### Withdrawal

The participation to this research project is purely voluntary and the participants are free to withdraw the study by asking the investigators of the study at any time.

### Description of measures taken to reduce and avoid bias

Subjects will be included chronologically and successively as long as they meet the inclusion/non-inclusion criteria and have given their consent to participate. The attrition bias is restricted as the monocentric nature of the study, the small number of subjects and the expertise of the staff involved in the study will limit the number of missing data. Data will be entered into the eCRF on an ongoing basis.

### Data collection

No medical procedures will be performed on the participants included in this study and no foreseeable risks are associated with this research. Stool samples do not entail any risk under the intended conditions of use. The provided collection kits and instructions enable samples to be taken simply, quickly and hygienically. The constraints are related to the stool collection and the time spent on the questionnaires. After the volunteers have been fully informed, consent will be obtained and appropriate procedures will be implemented for the processing of personal data.

Data will be entered using the Skezia software (https://skezia.io/) via the eCRFs by everyone involved in the project who has secure access to the eCRF. The Nutrition Investigation Unit will be responsible for entering clinical data. The data will be the property of the study sponsor. Anonymized data may be exported to the study directors.

All the information required by the protocol will be recorded in observation notebooks and an explanation will be given for any missing data. The data relating to the study that will be collected in the observation notebook will be considered as source data (Table 2).

**Table 2:** Overview of the data collected for each enrolled volunteer.

	Selection visit	Stool collection visit	final visit for 3 volunteers
Participant characteristics (age, gender)	X		
Anthropometric measures (weight, height and BMI)	X		X
health questionnaire	X		X
diet questionnaire	X		X
3- days dietary record		X	X
bristol stool scale for usual stool consistency		X	
Bristol stool scale for the donated stool		X	X

### The 24-hour dietary recall (24HR)

A 24-hour recall was used as a nutritional research tool for assessing individual's dietary intake over the previous 24-hour [43]. It involved the interview of individuals by a dietician to obtain detailed information about all foods and beverages ingested, from midnight to midnight, though main meals (breakfast, lunch, dinner) and outside mealtimes, including portion sizes and preparation methods.

### Dietary questionnaire for fiber consumption and selection criteria assessment

Spontaneous dietary fiber intake referred to the fiber consumed through dietary choices without intervention. The first step in assessing fiber intake was to compile a list of consumed foods based on the 24-hour dietary recall. This information was cross-referenced with the French food composition table CIQUAL 2020 [44] database to obtain the fiber content of each food item. Spreadsheets were used to organize and calculate the total fiber intake. Portion sizes were estimated using photographs from a validated picture booklet [45]. These portions sizes were applied to the corresponding food recorded in dietary recall, ensuring accuracy in quantifying fiber intake. During this interview, the following dietary criteria were also checked: diversified diet, rich in fruit and vegetables and low in ultra-processed foods.

## 3-Days food record

Before stool donation, dietary intake was estimated by means of three-day food records completed by the participants [43]. To remind participants to record all foods consumed, a food record was used including eight meal occasions (before breakfast, breakfast, morning snacks, lunch, afternoon snacks, evening meal, evening snacks, night snacks) referring to the current day. Participants had a face-to-face training in advance for the completion of the food records. Additionally, participants received instructions about the level of details required to describe foods and amounts consumed, including the name of the food, preparation methods, recipes for mixed foods and portion sizes. Portion sizes were reported in household measures, based on pictures or measured in gram or milliliters. During the interview with a dietician, the food record was checked to ensure an adequate level of details in describing foods and food preparation methods. Subsequently, each ingredient or food was translated into nutrients by use of CIQUAL French food composition table [45]. From these records, dietary intakes (in energy, macro- and micronutrients) were obtained using Nutrilog dietary software (https://nutrilog.com/).

### Sample size

To determine the minimum number of individuals required to achieve the project's objectives, and to provide an initial justification for our positive selection approach based on fiber consumption, we used healthy individuals from a published French cohort [46], representative of the French population we will target in the iTARGET study. The minimum number of individuals required to guarantee the detection of a species at least once was calculated for the19 selected species of interest (Table 1) using a binomial distribution with a probability of ≥ 0.95. Using this methodology, it was calculated that 4.4 ± 6.3 individuals were required to detect one of the 19 bacterial species of interest in the high-fiber diet group at least one time, while it would require 12,8 ± 34,1 individuals in the low-fiber group. Taking into account the mean and standard deviation, a minimum of 10 individuals on a high-fiber diet is required to maximize the prevalence of each of the selected microbial species of interest (Table 1).

### Data analysis and outcomes

Subjects will be included chronologically and successively as soon as they meet the inclusion criteria and have given their consent to participate. The monocentric nature of the study, the small number of subjects and the expertise of the UEN staff involved in the study will limit the number of missing data. Data will be collected using observation notebooks specific to the study, using the Skezia software with secure access (https://skezia.io/). The software allows quality control of the data and monitoring of modifications, with the date and name of the person who made the modifications.

Our primary outcome is the isolation and culture of at least one isolate of each species of interest, previously identified by metagenomic data analysis and literature review (Table 1). The metagenomic identification of additional bacterial species of interest will rely on the bioinformatical and biostatistical of the French Milieu Intérieur cohort [47] for which we have obtained the consortium's agreement for use within the strict framework of this project (see iTARGET in https://www.milieuinterieur.fr/en/about-us/collaborations/), and additional public shotgun metagenomic datasets [10].

The secondary outcomes will be to first obtain metagenomic profiles of the faecal microbiota using a high throughput shotgun sequencing strategy [48] in order to identify the participant carriers for bacteria of interest in relation to their dietary profiles [10,49,50]. Combined analysis of metagenomic data, health records and diet profiles will be performed under the R software (version 3.6) [51]. Second, we aim to acquire phenotypic and genetic and/or genomic characteristics of the isolates, including growth parameters, production of bacterial metabolites, general sugar metabolisms, 16S rDNA sequencing and functional potential through genome annotation. We will collect and characterize at least one strain per donor and per media for the selected species of interest (Table 1).

## Declaration of competing interest

The authors declare that they have no known competing financial interests or personal relationships that could have appeared to influence the work reported in this paper.

## Data Availability

No data was used for the research described in the article.
